# Phytotherapy known and applied by head-neck cancer patients and medical students to treat oral discomfort in Germany: an observational study

**DOI:** 10.1007/s00432-022-04200-0

**Published:** 2022-07-23

**Authors:** Maria-Louisa Ritschel, Jutta Hübner, Rebecca Wurm-Kuczera, Judith Büntzel

**Affiliations:** 1 Klinik für Innere Medizin II, Hämatologie und Internistische Onkologie, Universitätsklinikum Jena, Jena, Germany; 2 Klinik für Hämatologie und Medizinische Onkologie, Universitätsmedizin Göttingen, Göttingen, Germany; 3 Department of Hematology and Medical Oncology, University Medical Center, Robert-Koch-Str. 40, 37075 Göttingen, Germany

**Keywords:** German folk medicine, Phytotherapy, Head-neck cancer, Oral discomfort

## Abstract

**Background:**

Plant remedies are often used next to conventional standard of care by cancer patients. However, user rates are often underestimated and physicians usually feel not equipped to counsel patients. Hence, routinely recording the use of phytotherapy and sufficient knowledge on herbal medicine of the medical staff are required to improve the situation. Appraising the current state of knowledge of medical students may offer insight if education on herbals needs improvement. Here, we propose a simple anamnesis tool to assess knowledge and usage pattern of medical plants and demonstrate how to extrapolate symptom cluster participants associated with plants investigated in this study focussing on the common symptom of oral discomfort.

**Methods:**

By screening German literature (popular science, specialist’s literature, books for the interested layman) on medical plants used for treating oral discomfort, we were able to deviate a list of plants most often recommended for treating oral discomfort and to develop an anamnesis tool to assess knowledge and usage of 16 different plants. A group of 120 head-neck cancer patients (under surveillance, after receiving treatment) and 168 medical students were questioned at a collaborating out-patient clinic and via an online survey, respectively, in 2020. Students were additionally asked to write down indications of the plants they used in daily life. Knowledge and usage pattern were compared between both groups. Primary component analysis and heat-map analysis were used to visualize common and distinguishing features. Network analysis and VENN diagrams were used to extrapolate data of the medical students’ cohort.

**Results:**

Head-neck cancer patients and students show similar knowledge. However, students used significantly more plants in daily life than patients (*p* < 0.001). Overall, the user rate of patients and students were 82.50 and 93.94%, respectively. The top five most commonly known plants were similar in both groups (*Marticaria recutita L.*, *Zingiber offinicale ROSCOE, Taraxacum Wigg. Calendula officinalis L.*) with the exception of *Salvia officinalis L.* (patients’ cohort) and *Carum carvi/Cuminum cyminum L.* (students’ cohort). Despite this slight difference, usage pattern of the top five most commonly used plants was the same between the groups. Students’ indications were compared to indications found in the literature, unmasking several differences between commonly practiced and written knowledge on German phytotherapy. Network analysis revealed, that students associated certain plants with corresponding symptom clusters (e.g., coughing, oral mucositis).

**Conclusion:**

The majority of surveyed cancer patients as well as medical students use phytotherapy for treating oral discomfort. Both groups know and use similar plants. However, indications of written folk medicine differ from the lived and practiced tradition.

## Introduction

Next to surgery, radiation and chemotherapy are pillars of the oncological treatment for head-neck cancer (Pfister et al. 2020). Main site effects from radiation are (oral) mucositis, xerostomia, hypo- or dysgeusia and dentinal hypersensitivity (Skiba-Tatarska et al. 2016) and chemotherapy is also known to induce mucositis. A sufficient therapy of these symptoms is paramount as they lead to oral and oropharyngeal pain that requires opiate analgesics (Sonis 2013). Additionally, chemotherapy and targeted therapy lead to loss of appetite. Taken together, these symptoms of oral discomfort often prevent patients from eating normally and may be the cause for discontinuing therapy (Sonis 2013; Büntzel et al. 2019). 40 to 90% of all cancer patients use complementary and alternative medicine alongside to their oncological treatments. A major reason for doing so is the intention to alleviate side effects of cancer therapy (Molassiotis et al. 2005; Micke et al. 2009; Huebner et al. 2014; Wortmann et al. 2016). Herbal products are amongst the most commonly used approaches of complementary and alternative medicine (CAM) (Molassiotis et al. 2005; Huebner et al. 2014). A clinician should be able to advice patients on phytotherapy and CAM. However, a majority of physicians does not feel equipped to do so due to a lack of knowledge and a lack of evidenced-based, professional resources (Ventola 2010). The German National Guideline on Supportive Care and the National Guideline on Complementary and Alternative Medicine cover the four symptoms dysgeusia, loss of appetite, oral mucositis and (radiation-induced) xerostomia. However, only chamomile and aloe vera are mentioned as herbal agents used against oral mucositis and both Guidelines cite insufficient evidence for recommending either phytopharmakon as treatment option (Leitlinienprogramm Onkologie (Deutsche Krebsgesellschaft, Deutsche Krebshilfe, AWMF) 2020; Deutsche Krebsgesellschaft et al. 2021). However, if we wish to advice and guide patients using phytotherapy, we first have to assess, which plants are commonly used by patients before carefully searching literature databases for evidence plant by plant. At the same time, we should check, whether the knowledge on phytotherapy of physicians and patients is similar or if both stakeholders have different associations concerning plant use. Assessing medical students’ (and therefore future physicians’) knowledge and user behaviour offers a good compromise to approach this topic as the national competence-based catalogue of medical educational objective ensures a common base of learning objectives, including naturopathy/phytotherapy(Medizinischer Fakultätentag der Bundesrepublik Deutschland e. V. 2015). We previously screened literature for plants recommend for treating dysgeusia, loss of appetite, oral mucositis and xerostomia creating a hit list of the plants most often recommended by German folk medicine(Büntzel, Judith et al. 2020).

We here present a survey amongst head-neck cancer patients and medical students, assessing the knowledge and usage of 16 plants, that were recommended by German folk medicine for alleviating dysgeusia, loss of appetite, oral mucositis and xerostomia. Using a bioinformatics approach, we highlight common features between the knowledge and usage pattern of future medical doctors and cancer patients.

## Methods

### Questionnaire and recruitment

We screened ten German books on herbal (layman’s literature, specialist’s literature and plant identification books) for plants used for treating oral mucositis, xerostomia and loss of appetite. Plants were selected according to our previously published method: books addressing different groups of interest (specialist literature, popular science books, plant identification books) were screened to ensure a broad variety on information on German phytotherapy. Books used for screening literature are listed in (Table 1). If a plant was recommended for treating either oral mucositis, xerostomia or loss of appetite, they were recorded as herbal remedy. Each time a plant was recommended, a numerical point was assigned to the plant. The more often a plant was mentioned, the more points a plant gained. For each symptom, we generated a ‘hit list’ of plants (Buentzel et al. 2020; Büntzel et al. 2021, 2022). Then, the most often recommended plants for treating the symptoms were added to a simple questionnaire asking whether patients or medical students know of this plant (dichotomous answer “yes”/”no”). Patients or students indicating they knew the plant, were asked to answer, whether this plant was part of their everyday use (dichotomous answer “yes”/”no”). Use in daily life was defined as regular.

**Table 1:** Books screened

Books on phytotherapy screened for plants treating symptoms of oral discomfort
Achmüller, A (2012) Teufelskraut, Bauchwehblüml, Wurmtod: das Kräuterwissen Südtirols: Mythologie, Volksmedizin und wissenschaftliche Erkenntnisse, Edition Raetia, Bozen
Hensel W (2020) Welche Heilpflanze ist das?, 4th ed. Franckh Kosmos Verlag, Stuttgart
Landespflege, Bayerischer Landesverband f Gartenbau, Hohenberger E, Votteler W (2017) Gewürzkräuter und Heilpflanzen. 7th ed, Obst- und Gartenbauverlag des Bayerischen Landesverbandes für Gartenbau und Landespflege e.V, München
Mayer JG, Uehleke B, Saum PK (2013) Das große Buch der Klosterheilkunde, 1st ed. ZS Verlag Zabert Sandmann GmbH, München
Niederegger, O, Mayr C (2005) Hausbuch der Südtiroler Heilkraeuter Gesundheit aus der Natur, Athesia, Bozen
Pahlow AM (2004) Das große Buch der HEILPFLANZEN. Weltbild, Augsburg
Prentner, A (2017) Heilpflanzen der Traditionellen Europäischen Medizin: Wirkung und Anwendung nach häufigen Indikationen, Springer-Verlag, Berlin
Rätsch, C (2014) Heilpflanzen der Antike: Mythologie, Heilkunst und Anwendung, AT Verlag, Aarau
Stange R, Kraft K (2009) Lehrbuch Naturheilverfahren, 1st ed. Hippokrates, Stuttgart
Steigerwald P-A (2015) Phytotherapie pocket, 3rd ed. Börm Bruckmeier, Grünwald

Patients were recruited amongst the out-patients of the Department of Otolaryngology and Head Neck Surgery (Südharz Hospital, Nordhausen, Germany) between August and September 2020. Patients were visiting for follow-up care. All patients underwent chemotherapy and/or local radiation previously and therefore knew and had experienced oral discomfort. Visual aids containing images of the plants survey were used as supporting material, enabling patients either to recognize the plant by name, by image or by the context/indication the herbals were recommended by folk medicine. Patients requiring aid for participation were supported the medical staff.

Medical students were recruited via social media and both students of pre-clinical and clinical courses were allowed to participate. Students were questioned in their role as users of phytotherapy. The online tool https://soscisurvey.de was used for generating and hosting the survey. The online questionnaire was open for two months in 2020 (March and April). We used the same questionnaire for patients and students. However, the students’ survey had the additional feature: if students used one of the listed plants in daily life, they were asked to write down which indication they associated with this plant. Free text answers containing the students’ indications for plants surveyed were later on compared with the plants’ indications cited by literature.

This study was approved as a part of a larger research project by the local ethic committee of the medical faculty in Jena (approval numbers: 2020–1866-Bef, 2020–1881-Bef).

### Statistical analysis

Statistical analysis was conducted using Microsoft Excel 2016 and GraphPad (Version 8, GraphPad Software Inc., San Diego CA, US). Median and interquartile range was used to describe the number of plants known and used by patients and students. Rates of knowledge/usage were calculated as following:

$$Rate\,\,of\,\,knowlegde\,\,{\mkern 1mu} = \,\,{\mkern 1mu} \frac{{N\left[ {participants\,\,knowing\,\,plant\,\,X} \right]}}{{N\left[ {participants} \right]}}$$

$$Rate\,\,of\,\,usage\,\,{\mkern 1mu} = \,\,{\mkern 1mu} \frac{{N\left[ {participants\,\,using\,\,plant\,\,X} \right]}}{{N\left[ {participants} \right]}}$$

Number of plants known and used by patients and students were compared using an unpaired student’s *t*-test. A *p*-value < 0.05 was considered significant.

Heat-map cluster analysis and primary component analysis (PCA) were calculated for plants and indications surveyed as proposed in (Büntzel et al. 2021) using the online tool ClustVis (Metsalu and Vilo 2015). First, we used primary component analysis to e.g., compare answers of our two cohorts (patient vs. student cohort). Heat-maps were used for further visualization. VENN diagrams were drawn using the free software http://bioinformatics.psb.ugent.be/webtools/Venn/.

Free text answers of students were used for network analysis. Here, we first defined symptom complexes. Indications of single plants were sorted by symptom and symptom complex. Network analysis and subsequent visualization were conducted using https://ezlinavis.dracor.org/.

## Results

### Knowledge and usage pattern of herbal remedies of head-neck cancer patients

For this study we were able to include 120 head-neck cancer patients; clinical characteristics are listed in (Table 2). Patients know in median 8 [IQR 5.25–11.0] plants. The five most commonly known plants were *Marticaria recutita L.* (96.7%), *Zingiber offinicale ROSCOE* (92.5%), *Salvia officinalis L.* (91.7%), *Taraxacum Wigg.* (83.3%) and *Calendula officinalis L.* (73.3%). Table 3 shows a hit list of patients’ knowledge of all medical plants included in this study. Out of all 120 patients returning our questionnaire 82.50% confirmed using one of the listed medical herbs. In our cohort, patients used in median 3.0 [IQR 1.0–4.0] plants. The user rate was as followed: *Matricaria recutita L.* (70.0*%), Salvia officinalis L.* (57.5%), *Zingiber officinale ROSCOE* (46.7%), *Carum carvi/Cuminum cyminum L.* (25.0%) and *Calendula officinalis L.* (24.2%); for a list of the usage pattern of all plants, refer to (Table 3). Amongst the top five plants most often used by head-neck cancer patients are three plants commonly recommended for treating oral mucositis.

**Table 2:** Clinical characteristics patients’ cohort and demographic data students’ cohort

Clinical characteristics patients		
	Total	120
Gender	Male (*N*)	93
	Female (*N*)	27
Age	Mean ± SD [years]	65.61 ± 10.31
Cancer entity	Hypopharyngeal cancer (*N*)	7
	Laryngeal cancer (*N*)	37
	Nasal cavity cancer (*N*)	16
	Oral cavity cancer (*N*)	18
	Others (*N*)	12
	Salivary gland tumour (*N*)	7
	Tonsil cancer/ oropharyngeal cancer (*N*)	23
Demographic data students		
	Total (*N*)	168
Gender	Male (*N*)	45
	Female (*N*)	116
	Divers (*N*)	1
	n. i. (*N*)	6
Degree programme	Human medicine (*N*)	151
	Dental medicine (*N*)	1
	Others (*N*)	10
	n. i. (*N*)	6
Level of education	Pre-clinical course (*N*)	65
	Clinical course (*N*)	78
	Elective year (*N*)	11
	Others (*N*)	7
	n. i. (*N*)	7

*SD* standard deviation, *n. i. *no information

**Table 3:** Knowledge and user rates of patients and medical students

	Knowledge		Usage	
	Patients (%)	Students (%)	Patients %)	Students (%)
*Althaea officinalis L.*	27.0	25.0	3.0	0.7
*Angelica archangelica L.*	18.0	28.5	0.0	0.7
*Artemisia absinthium L.*	46.0	55.6	8.0	7.6
*Calendula officinalis L.*	73.0	82.6	24.0	17.4
*Centaurium Hill*	13.0	14.6	1.0	0.0
*Cetraria islandica L.*	18.0	50.7	8.0	21.5
*Cichorium intybus L.*	21.0	0.0	22.9	0.0
*Carum carvi et Cuminum cyminum*	63.0	88.2	25.0	28.5
*Gentiana lutea L.*	65.0	61.1	6.0	4.2
*Matricaria recutita L.*	97.0	95.1	70.0	54.9
*Malva sylvestris et neglecta*	52.0	46.5	12.0	4.2
*Plantago lanceolata L.*	58.0	63.9	10.0	11.1
*Potentilla erecta L.*	15.0	29.9	0.0	0.7
*Salvia officinalis L.*	92.0	93.1	58.0	44.4
*Taraxacum officinale Wigg*	83.0	86.8	9.0	8.3
*Zingiber officinale Roscoe*	93.0	95.8	47.0	56.9

### Knowledge and usage pattern of herbal remedies of German medical students

151 medical students, one dental student and ten participants stating “other” (6 students of physiotherapy, 4 medical staff) participated in our online survey about medical herbs (characteristics of participants are listed in Table 2). Three participants gave no information on educational status. Students know in median 8 [IQR 6.0–10.5] plants. The most commonly known plants are *Taraxacum Wigg.* (82.4%), *Carum carvi/Cuminum cyminum L.* (75.8%), *Calendula officinalis L.* (73.8%), *Zingiber officinale ROSCOE* (72.1%) and *Matricaria recutita L.* (70.9%). The rates of knowledge of all medical plants may be found in (Table 3). 93.9% off students asked use at least one of the 16 plants of our questionnaire in daily life. They use in median 4.0 [IQR 2.0–5.0] medical plants. The most commonly used plants are: *Zingiber officinale ROSCOE* (78.8%), *Matricaria recutita L.* (78.2*%), Salvia officinalis L.* (66.7%), *Carum carvi/Cuminum cyminum L.* (38.8%) and *Calendula officinalis L.* (26.1%). The usage pattern of all plants is listed in Tale 3.

### Comparing knowledge and usage pattern of head-neck cancer patients and medical students

**Fig. 1:**
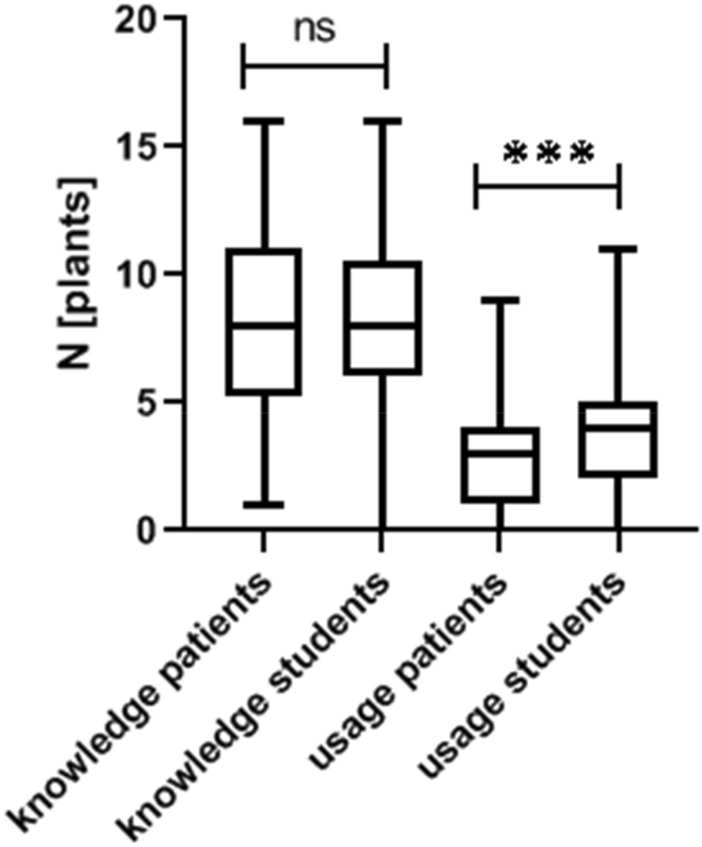
Knowledge and usage pattern of patients and medical students. While both cohorts show a similar knowledge pattern, students use a significantly higher number of plants in daily life (unpaired student’s *t*-test, *p* < 0.001)

While we did not observe a difference between the number of plants known to medical students and patients, medical students use significantly more medical herbs in daily life (*p* < 0.001, *t* = 3.436, Student’s *t*-test, Fig. 1).

While in both groups *Marticaria recutita L.*, *Zingiber offinicale ROSCOE, Taraxacum Wigg.* and *Calendula officinalis L.* are amongst the top five commonly known plants, *Salvia officinalis L.* and *Carum carvi/Cuminum cyminum L.* were only amongst the top five amongst patients and students respectively. However, both patients and students have the same usage pattern of medical plants when looking at the five most commonly used herbs (*Calendula officinalis L., Carum carvi/Cuminum cyminum L., Matricaria recutita L., Salvia officinalis L.* and *Zingiber officinale ROSCOE*).

**Fig. 2:**
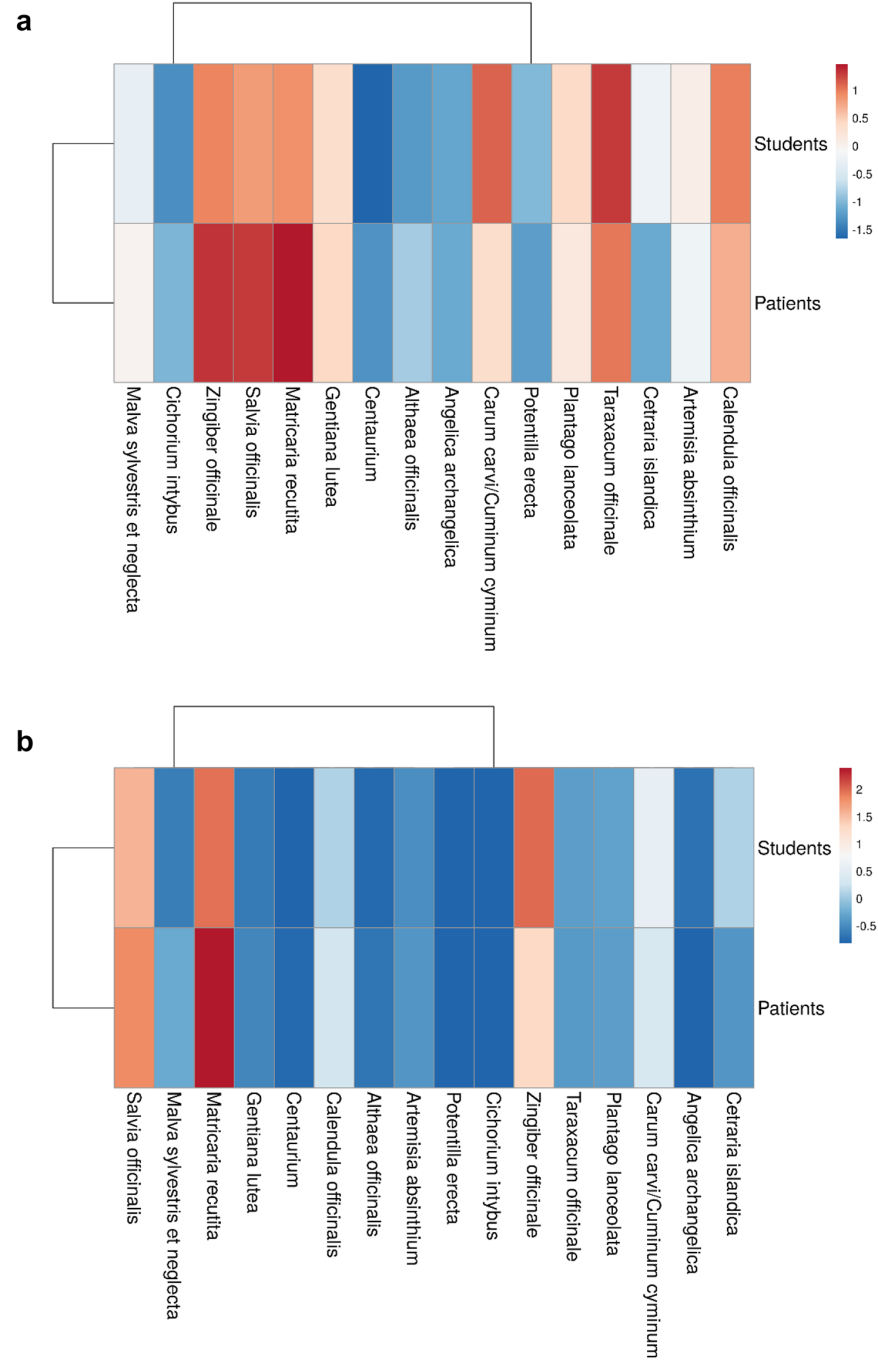
Visualization of knowledge and usage pattern of patients and medical students. Red indicates a high percentage of either knowledge or usage, blue a low percentage of knowledge and application in daily life. **a** The overall knowledge pattern of both cohorts is similar. While well known in both groups, *Matricaria recutita L*., *Salvia officinalis L.* and *Zingiber officinale Roscoe* show a higher percentage of knowledge in the patient cohort. **b** Usage pattern uncovers a preference of patients for *Matricaria recutita L.*, while students are more prone to use *Zingiber officinale Roscoe* in daily life

Multivariate clustering analysis shows the following: there are differences in the rate of how known each of the 16 medical herbs are. However, both patients and students show a at least similar pattern of familiarity with the different phytodrugs (Fig. 2a). Only *Cetraria islandica L.* and *Carum carvi/Cuminum cyminum L.* are an exception being more commonly known to medical students. When looking at the general usage pattern of herbs (Fig. 2b), both groups again align, however, head-neck cancer patients show a focus on *Matricaria recutita L.* and *Salvia officinalis L.*, two plants commonly recommended by folk medicine for treating oral mucositis(Buentzel et al. 2020).

### Comparing knowledge of phytotherapy literature with knowledge of medical students

**Fig. 3:**
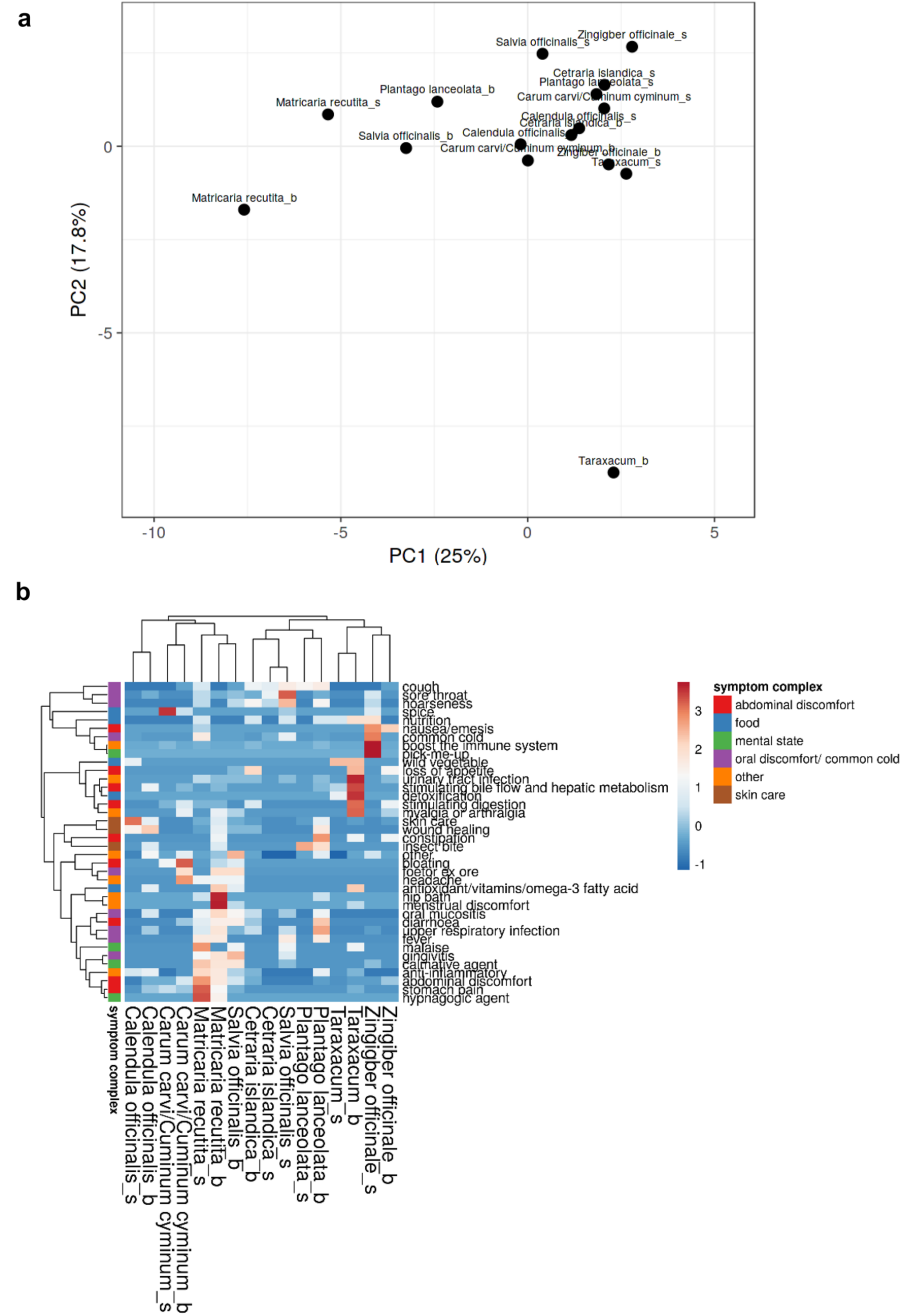
Visualizing common features and differences between written and oral knowledge on the indications of medicinal plants. Plant names are marked with a “b” and “s” indicating indications taken from books and students’ indications respectively **a** Primary component analysis and **b** heat-map analysis show common features between written (literature) and oral knowledge in plants like *Calendula officinalis L.* or *Cetraria islandica* L. Divergent indications are e.g., observed for *Salvia officinalis L.* Red indicates a high percentage of either knowledge or usage, blue a low percentage of knowledge

Knowledge on what plant to use for treating a specific symptom is preserved in different ways. But does the knowledge found in literature (be it layman’s or specialist’s literature) correspond to people’s “everyday” knowledge? Students were asked to write down for which indication they use a plant out of the 16 proposed by us. Overall, students wrote down indications for these eight plants: *Calendula officinalis L.*, *Cetraria islandica L.*, *Carum carvi/Cuminum cyminum L.*, *Matricaria recutita L.*, *Plantago lanceolate L.*, *Salvia officinalis L.*, *Taraxacum Wigg.* and *Zingiber offinicale ROSCOE*. Indications for using these plants cited by literature (layman’s and specialist’s literature) were assessed for each plant. Indications for using these medical plants were categorized by symptom and symptom complex as stated above. Taken together, we had to define 37 items (indications) to compare students’ and literature’s knowledge. A list of all indications is listed in (Table 4). We used primary component analysis and heat-mapping for comparing both groups. PCA shows that literature’s and students’ indications of *Calendula officinalis L., Cetraria islandica L.* and *Carum carvi/Cuminum cyminum L.* cluster closely together, while there are more differences when looking at e. g. *Matricaria recutita L.*, *Salvia officinalis L.* or *Zingiber offinicale ROSCOE*. However, indications for using *Taraxacum Wigg.* seem to highly differ when comparing indication ascribed by literature or students (Fig. 3a). Heat-mapping explains partly the observed differences of the PCA plot: while students know that *Taraxacum Wigg.* might be used as (edible) wild vegetable, they know less or nothing about the plant’s other various indications- e.g., using the plant for improving digestion or treating loss of appetite. Heat-mapping also unmasks other differences in knowledge: many students explained, that *Zingiber officinale ROSCOE* was useful to improve/support the immune system—an indication not found in literature. On the other hand, several indications for plants found in literature were not (widely) known to medical students: e.g,. using *Matricaria recutita L.* for treating (menstruation) cramps, *Plantago lanceolata L.* or *Saliva officinalis L.* for treating different symptoms of oral mucositis (Fig. 3b).

**Table 4:** Symptoms stated by students

List indications (students/books)	Symptom cluster
Common cold	Oral discomfort
Cough	Oral discomfort
Foetor ex ore	Oral discomfort
Ginigivitis	Oral discomfort
Hoarseness	Oral discomfort
Oral mucositis	Oral discomfort
Sore throat	Oral discomfort
Upper respiratory tract infection	Oral discomfort
Stomach pain	Abdominal discomfort
Abdominal discomfort	Abdominal discomfort
Stimulating digestion	Abdominal discomfort
Constipation	Abdominal discomfort
Diarrhoea	Abdominal discomfort
Nausea/emesis	Abdominal discomfort
Bloating	Abdominal discomfort
Loss of appetite	Abdominal discomfort
Stimulating bile flow and hepatic metabolism	Abdominal discomfort
Insect bite	Skin care
Skin care	Skin care
Wound healing	Skin care
Malaise	Mental health
Calmative agent	Mental health
Hypnagogic agent	Mental health
Pick-me-up	Mental health
Antioxidant/vitamin/omega-3 fatty acid	Food
Nutrition	Food
Spice	Food
Anti-inflammatory	Other
Boosting the immune system	Other
Detoxification	Other
Fever	Other
Headache	Other
Hip bath	Other
Menstrual discomfort	Other
Myalgia or arthralgia	Other
Other	Other
Urinary tract infection	Other

### Analysing and uncovering common indications of medical plants described by literature and medical students

**Fig. 4:**
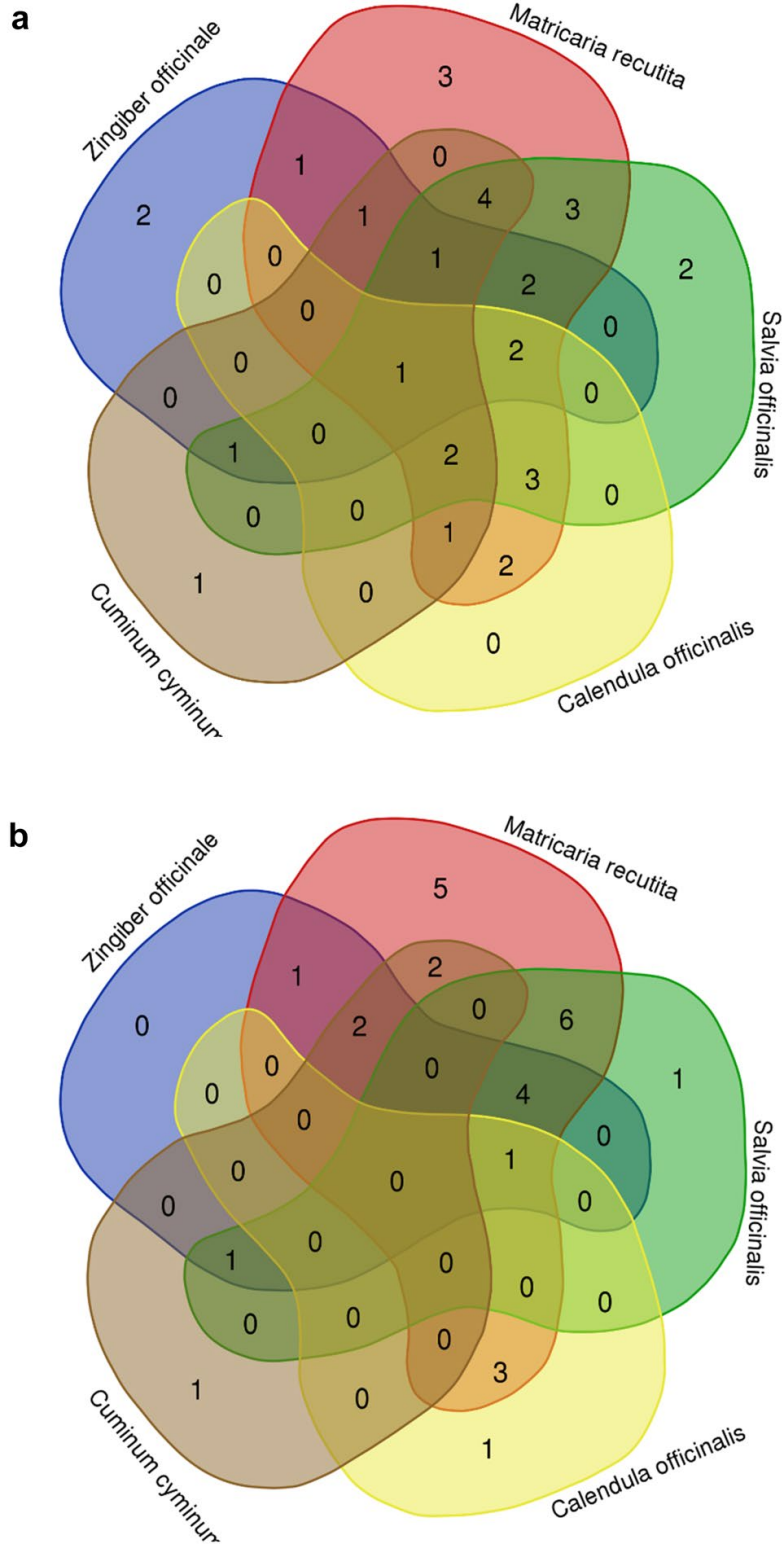
VENN diagrams of *Calendula officinalis L*., *Carum carvi L./ Cuminum cyminum L.*, *Matricaria recutita L.*, *Salvia officinalis L.* and *Zingiber officinale Roscoe* show N(overlapping features) between these plants found in **a** literature and **b** as indicated by medical students

Both literature and medical students described usually more than one indication for using medical herbs. We chose the five plants most often used by medical students in daily life (*Calendula officinalis L.*, *Carum carvi/Cuminum cyminum L.*, *Matricaria recutita L.*, *Salvia officinalis L.*, *Zingiber officinale ROSCOE*) and set out to compare whether these share common indications using VENN diagrams. We analysed indications given by students and indications extracted from literature. While analysing indications found in literature, the VENN-plot (Fig. 4a) uncovered a common feature of all five plants: the application for abdominal discomfort. This common feature was not described by medical students. Here, students identified *Calendula officinalis L.*, *Matricaria recutita L.*, *Salvia officinalis L.* and *Zingiber officinale ROSCOE* as plants used for treating the common cold (Fig. 4b). Other shared indications for combinations of less than five (literature indications) or respectively four (medical student indications) plants are listed in (Table 5).

**Table 5:** Indications stated by literature and students

Indications in literature		
N [plants]	Plant designation	Common indcation(s)
5	*Calendula officinalis L.*, *Cuminum cyminum L.*, *Matricaria recutita L.*, *Salvia officinalis L.*, *Zingiber officinale ROSCOE*	Abdominal discomfort
4	*Calendula officinalis L, Matricaria recutita L.*, *Salvia officinalis L.*, *Zingiber officinale ROSCOE*	Sore throat skin care
4	*Cuminum cyminum L.*, *Matricaria recutita L.*, *Salvia officinalis L.*, *Zingiber officinale ROSCOE*	Myalgia or arthralgia
4	*Calendula officinalis L.*, *Cuminum cyminum L.*, *Matricaria recutita L.*, *Salvia officinalis L*	Stomach pain anti-inflammatory
3	*Matricaria recutita L.*, *Salvia officinalis L.*, *Zingiber officinale ROSCOE*	Common cold urinary tract infection
3	*Cuminum cyminum L.*, *Matricaria recutita L.*, *Zingiber officinale ROSCOE*	Stimulating digestion
3	*Cuminum cyminum L.*, *Salvia officinalis L.*, *Zingiber officinale ROSCOE*	Cough
3	*Calendula officinalis L.*, *Matricaria recutita L.*, *Salvia officinalis L.*,	Antioxidant/vitamins/omega-3 fatty acid wound healing oral mucositis
3	*Cuminum cyminum L.*, *Matricaria recutita L.*, *Salvia officinalis L*	Bloating headache foetor ex ore diarrhoea
3	*Calendula officinalis L.*, *Cuminum cyminum L.*, *Matricaria recutita L.*,	Stimulating bile flow and hepatic metabolism
2	*Matricaria recutita L.*, *Zingiber officinale ROSCOE*	Constipation
2	*Matricaria recutita L.*, *Salvia officinalis L*	Calmative agent menstrual discomfort gingivitis
2	*Calendula officinalis L.*, *Matricaria recutita L.*,	Upper respiratory infection insect bite
1	*Zingiber officinale ROSCOE*	Nausea/emesis loss of appetite
1	*Matricaria recutita L*	Fever hip bath hypnagogic agent
1	*Salvia officinalis L*	hoarseness malaise
1	*Cuminum cyminum L*	Spice
Indications according to medical students’ knowledge		
4	*Calendula officinalis L.*, *Matricaria recutita L.*, *Salvia officinalis L.*, *Zingiber officinale ROSCOE*	Common cold
3	*Matricaria recutita L.*, *Salvia officinalis L.*, *Zingiber officinale ROSCOE*	Calmative agent hoarseness sore throat upper respiratory infection
	*Cuminum cyminum L.*, *Matricaria recutita L.*, *Zingiber officinale ROSCOE*	Boost the immune system abdominal discomfort
3	*Cuminum cyminum L.*, *Salvia officinalis L.*, *Zingiber officinale ROSCOE*	Spice
2	*Matricaria recutita L.*, *Zingiber officinale ROSCOE*	Nausea/emesis
2	*Matricaria recutita L.*, *Salvia officinalis L*	Fever malaise gingivitis cough oral mucositis urinary tract infection
2	*Calendula officinalis L.*, *Matricaria recutita L*	anti-inflammatory skin care wound healing
2	*Cuminum cyminum L.*, *Matricaria recutita L*	Stomach pain stimulating digestion
1	*Matricaria recutita L*	Pick-me-up headache hip bath diarrhoea hypnagogic agent
1	*Salvia officianlis L*	Nutrition
1	*Calendula officinalis L*	Wild vegetable
1	*Cuminum cyminum L*	Bloating

**Fig. 5:**
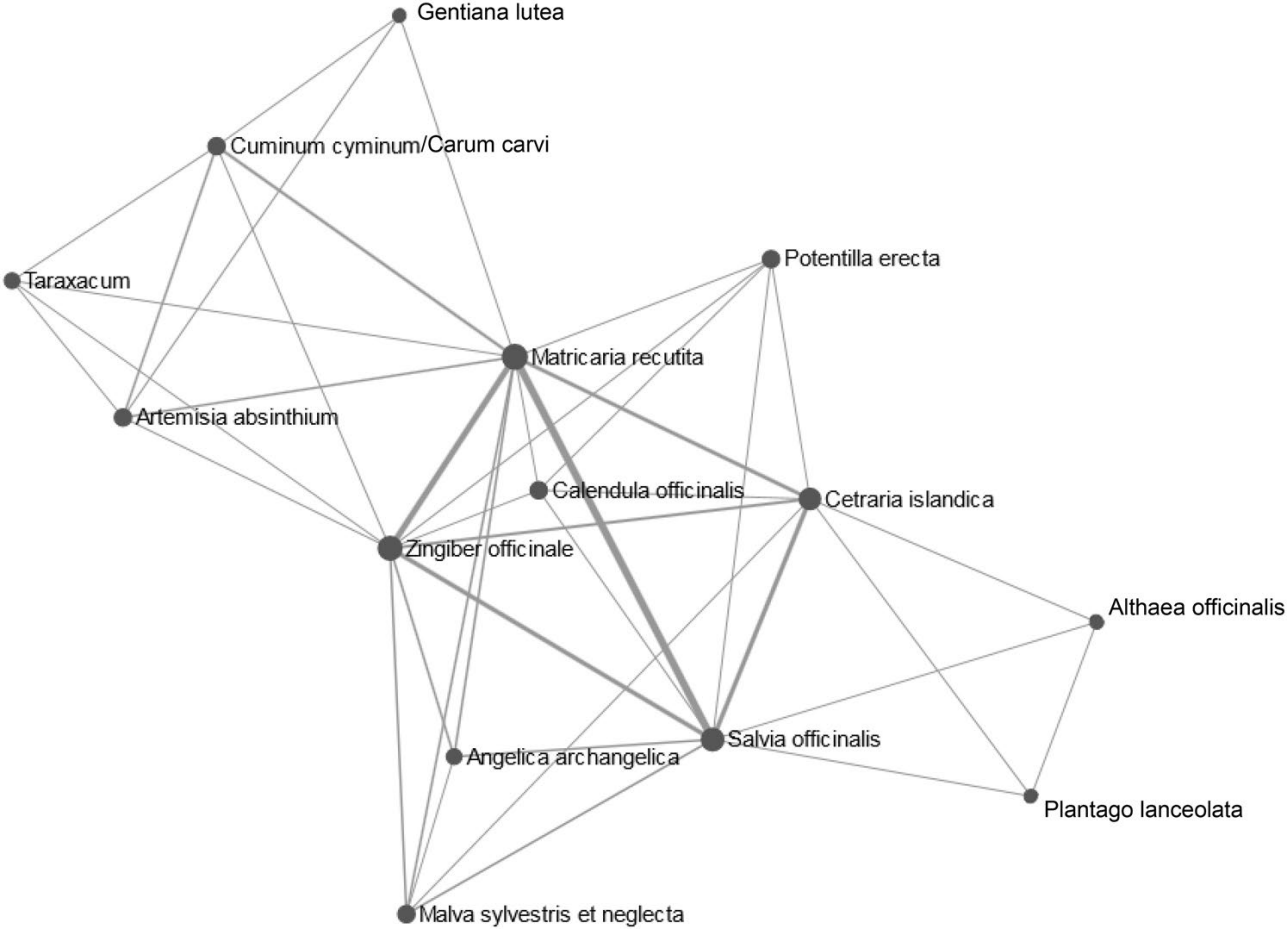
Network analysis of plants for which students stated at least one indication visualizing common indications and relationships between plant. Three larger clusters are identified corresponding with the symptom complexes “abdominal discomfort”, “oral discomfort” and “coughing”. Plants belonging to more than one cluster are easily recognized (e.g., *Matricaria recutita L.* belonging to both “abdominal discomfort” and “oral discomfort”

As students offered a large variety of potential indications for plants, we used a network tool to demonstrate common indications and relationships between plants. Here, the herbals cluster in three groups that correspond to common symptom complexes: *Gentiana lutea L.*, *Carum carvi/Cuminum cyminum L.*, *Taraxacum Wigg.* and *Arthemisa absinthum L.* form a group of plants used for abdominal discomfort. *Matricaria recutita L.* and *Zingigber officinale ROSCOE* are located at the edge of this cluster while also belonging to the cluster of plants used for oral discomfort (*Matricaria recutita L.*, *Zingiber officinale ROSCOE*, *Potentilla erecta L.*, *Calendula officinalis L.*, *Angelica archangelia L.*, *Malva sylvestris et neglecta*, *Cetraria islandica L.* and *Salvia officinalis L.*). In parallel to the left outer cluster “abdominal discomfort”, we observe a third cluster of herbals used against “cough” consisting of *Althaea officinalis L.* and *Plantago lanceolacta L.* as well as two plants (*Salvia officinalis L.* and *Cetraria officinalis L.*) that also belong to the “oral discomfort” cluster (Fig. 5). Here, network analysis uncovers indication/symptom clusters that medical students associated with the different plants, visualizing common and different features of the herbals appraised in our survey.

## Discussion

Concerning phytotherapy, different studies reported user rates ranging from 19.7 to 38% in cancer patients(Engdal et al. 2008; Afifi et al. 2010; Damery et al. 2011; Lima et al. 2018), while we describe user rate of 82.5% in patients that returned questionnaire. This shows a number twice as high as in most publications assessing the usage of phytotherapy in cancer patients.

However, one should keep in mind that (1) patients do not always communicate their use of phytotherapy with their treating oncologist(Planta et al. 2000; Micke et al. 2009) and (2) herbs may not always considered to be medications by our patients. Concerning the high user rates of herbal remedies in our survey, studies amongst other (non-cancer patient) populations yielded user rates of > 50% up to nearly 90% (Planta et al. 2000; Green et al. 2017). Older patients and women are more likely to use CAM and phytotherapy (Boing et al. 2019), yet we also observed a high user rate amongst medical students, belonging to the younger age (and healthy) cohort.

### Canon of medical plants for treating the most common symptoms of head-neck cancer patients

Despite the different focus of students and patients, at the end we observe a similar knowledge and usage pattern between these two groups. The most commonly applied plants were *Calendula officinalis L.*, *Carum carvi L./Cuminum cyminum L.*, *Matricaria recutita L.*, *Saliva officinalis L.*, *Zingiber officinalis Roscoe*. While we started with 16 most recommended plants, our findings condensed our initial, literature-based canon of medical plants very quickly to a much smaller number of five plants. Moreover, our results show the limits of only screening literature for compiling lists of the plants most often recommended for treating a certain symptom. While a plant like *Potentilla erecta L.* was amongst the most often recommended herbs in literature, very few patients or students even knew of this medical plant. We show that written traditional knowledge on phytotherapy is not always congruent with actual practised application of medical plants. Our initial ‘hit lists’ of medical plants was derived from books showing a broad variety and richness of herbals traditionally used for treating oral discomfort. However, we also should consider practicability: (1) only a small part of plants proposed by literature are in use, however those are well known. (2) a common base of knowledge between patients and medical students, the future generation of physicians, facilitates understanding and communication. (3) a reduction from our initial 16 plants to five simplifies checking for potential interactions with oncological therapy or interactions and 4) also narrows the focus for future systematic reviews or meta-analyses. Amongst the five plants above are three- *Calendula officinalis L.*, *Matricaria recutita L.* and *Salvia officinalis L.*- for which we already have clinical trials with evidence supporting the usage of these plants for treating symptoms of oral mucositis(Buentzel et al. 2020). Furthermore, a small pilot study showed, that the application of Zingiber officinale Roscoe increased the rate of salivation in head-neck cancer patients (Shooriabi et al. 2016). All together, we have clinical evidence (of varying degree) for four out of five plants used by our patients. This may be the first step to enable medical staff, that often feels not equipped to advice patients (Ventola 2010), to give counsel. Further, teaching this existing evidence-based knowledge and integrating it into the communication between medical staff and patients would be an improvement of the current situation (refer to (Leitlinienprogramm Onkologie (Deutsche Krebsgesellschaft, Deutsche Krebshilfe, AWMF) 2020; Deutsche Krebsgesellschaft et al. 2021)).

### Applying health-informatical tools to make knowledge on phytotherapy visible

The application of methods usually used for interpreting large biological datasets offers a novel approach for analysing our ethnobotanical research problem enabling us to get new insights into the knowledge base of different stakeholder groups.

As described in the previous paragraph written (folk medicine) and commonly practiced (indications stated by our students) knowledge on how to use a specific medical plant differ as easily as the knowledge and usage pattern of different stakeholder groups (patients, medical students). Using heatmaps to compare the knowledge and the usage pattern of patients and medical students, we were able to highlight common features between groups e. g. similar usage rates of *Calendula officinalis L., Carum carvi/Cuminum cyminum L., Matricaria recutita L., Salvia officinalis L.* and *Zingiber officinale ROSCOE*. However, heatmaps also quickly uncover distinguishing features between patients and medical students e.g., differences in the knowledge pattern of medical students namely for *Cetraria islandica L.* and *Carum carvi/Cuminum cyminum L.*

Both PCA and heatmaps are not only useful to compare the behaviour of patients and medical students. These methods also enable as to compare different pools of knowledge. We applied PCA and heatmap to our indication datasets (books’ indications, medical students’ indications—free text answers). We extrapolated common and distinguishing features between those datasets: for example, the heat-map showed that two herbs usually recommended by literature for treating symptoms of oral mucositis-*Plantago lanceolata L.* and *Saliva officinalis-* were not known at all to medical students. PCA on the other hand enables us to analyse how similar written and commonly practiced indications of a single plant are. While commonly practiced and written knowledge seem congruent for *Calendula officinalis L.*, we observe differences regarding *Taraxacum Wigg.* or *Matricaria recutita L.* This incongruence in indications can not only be observed comparing written (folk medicine) indications with commonly practiced (students’) indications, but also when comparing official indication mandated by the German Federal Institute for Drugs and Medical Devices. So, if the students’ knowledge on indications is neither sufficiently based on official indications (Büntzel et al. 2022) or, as shown here, on indications as supplied by written folk medicine, we should carefully check: What is actually the knowledgebase of this generation of future physicians? We observed only partly an overlap with written folk medicine. Now, we also need to consider that students’ and patients’ knowledge and usage are quite similar and could be described as “lived” and practiced folk medicine in Germany. This distinction is quite important, as those students may base their future counselling of patients on their own experiences (folk medicine) rather than evidence-based medicine.

### Applying health-informatical tools to make common indications of medical plants visible

In a second step we asked ourselves how to visualize common features of different plants. VENN diagrams offer an overview up to five plants, identifying the common indication “abdominal discomfort” for C*alendula officinalis L.*, *Carum carvi/Cuminum cyminum L.*, *Matricaria recutita L.*, *Salvia officinalis L.* and *Zingiber officinale ROSCOE* in German literature. If we wish to analyse relationships and common indications between more than five plants however, VENN diagrams do not cover all aspects. Network analysis of the indications stated by medical students sorted plants according to symptom clusters and was able to highlight plants with indications falling into several clusters: e.g., *Matricaria recutita L.* or *Zingiber officinale ROSCOE* both belonging to the oral and abdominal discomfort cluster. At the same time network analysis revealed a new cluster (“coughing”). Taken together both VENN diagrams and network analysis are good tools to visualize relationships and common features of indications of medical plants.

### Limitations and opportunities

As popular science books usually do not use Latin plant designations, names for the same plant may vary. The authors cross-referenced names, however regional differences in naming plants (e.g., “Malve” vs. “Käsepappel”) may have resulted in an underestimation of those. To ensure specificity and clarity we stuck to only books published in the authors native language (German). While we screened ≥ ten German phytotherapy books per symptom, we have to consider that this selection might be biased due to this restriction. However, we are the first to present a list the most commonly recommended herbs used for treating dysgeusia, loss of appetite, oral mucositis and xerostomia in the German-speaking part of Europe. In favour of assessing traditional indications of German folk medicine, the officially mandated indications of the BfArM were ignored. The method we first proposed in (Buentzel et al. 2020) allows to get an overview over written folk medicine. However, as we demonstrate here, cross-referencing via a survey is necessary to ascertain which plants are actually in use. We only chose two populations to assess knowledge and usage pattern of the 16 plants surveyed. Head-neck cancer patients were chosen due to the high frequency of oral discomfort during cancer treatment in this population. Medical students were assessed, to get an idea how deep the knowledge on phytotherapy is in this population of future physicians. A follow-up study should assess whether the knowledge und usage pattern of head-neck cancer patients is transferrable to a larger cohort of cancer patients suffering from different entities.

## Conclusions

Head-neck cancer patients and medical students share a common knowledge base on phytotherapy. User rates are high (> 80%) in both groups. Informatical tools like PCA and heatmap allow to compare knowledge bases and behaviours of patients and medical students alike. Further, these informatical methods are useful to extrapolate differences and common features in larger datasets on indications derived from literature and medical students.
